# The Analysis of Neutrophil to Lymphocyte Ratio (NLR), Lymphocyte to Monocyte Ratio (LMR), and Platelet to Lymphocyte Ratio (PLR) in Patients with Adenoidectomy and Tonsillectomy

**DOI:** 10.3390/jcm13185457

**Published:** 2024-09-14

**Authors:** Jeong-Mi Kim, Jeong-Seok Choi

**Affiliations:** 1 Department of Otorhinolaryngology, Head and Neck Surgery, College of Medicine, Inha University, 27 Inhang-ro, Jung-gu, Incheon 22332, Republic of Korea; jeongmi77@gmail.com; 2 Research Center for Controlling Intercellular Communication (RCIC), College of Medicine, Inha University, 100 Inha-ro, Michuhol-gu, Incheon 22212, Republic of Korea; 3 Program in Biomedical Science & Engineering, Department of Biomedical Science, Inha University, 100 Inha-ro, Michuhol-gu, Incheon 22212, Republic of Korea

**Keywords:** neutrophil to lymphocyte ratio (NLR), lymphocyte to monocyte ratio (LMR), platelet to lymphocyte ratio (PLR), adenoidectomy, tonsillectomy

## Abstract

**Background**/**Objectives:** Adenoidectomy and tonsillectomy are among the most commonly performed procedures in ENT practice. The neutrophil-to-lymphocyte ratio (NLR), lymphocyte-to-monocyte ratio (LMR), and platelet-to-lymphocyte ratio (PLR) are recognized inflammatory markers. This study aims to evaluate the changes in NLR, PLR, and LMR in patients undergoing adenoidectomy and tonsillectomy. **Methods**: The study group consisted of 980 patients who underwent adenoidectomy and/or tonsillectomy. Preoperative and postoperative inflammatory markers were measured in all patients. The NLR, LMR, and PLR values were then calculated and analyzed. **Results:** In patients undergoing adenoidectomy and/or tonsillectomy, the postoperative NLR was significantly lower than the preoperative NLR. Similarly, the postoperative LMR was significantly higher, and the postoperative PLR was significantly lower compared to their preoperative values. **Conclusions:** The significant changes in NLR, LMR, and PLR following adenoidectomy and/or tonsillectomy suggest a reduction in systemic inflammation post-surgery. These findings indicate that these procedures may contribute to the improvement of inflammatory status in patients, highlighting the potential role of these markers in monitoring surgical outcomes.

## 1. Introduction

Tonsillectomy and adenoidectomy are among the most commonly performed procedures in ENT practice [1,2]. Tonsil and adenoid hypertrophy can lead to upper airway obstruction, resulting in alveolar hypoventilation, which may cause chronic hypoxia and hypercapnia [3,4]. Chronic hypoxia, in turn, can lead to pulmonary arterial hypertension, cor pulmonale, and eventually decompensated heart failure [5,6]. Importantly, these severe outcomes have been found to be reversible with adenoidectomy, highlighting the significance of this treatment [7,8].

Adenoidectomy and tonsillectomy are among the most frequently performed surgical procedures in otolaryngology (ENT) practice, often indicated for conditions such as recurrent tonsillitis, obstructive sleep apnea, and chronic adenoiditis. These procedures are particularly common in pediatric populations but are also performed in adults for various indications. The primary goal of adenoidectomy and tonsillectomy is to alleviate symptoms associated with tonsil and adenoid hypertrophy, which can lead to significant morbidity, including airway obstruction, recurrent infections, and sleep disturbances [9,10].

In recent years, there has been increasing interest in the use of hematologic inflammatory markers as potential indicators of systemic inflammation and disease severity in various clinical settings [11]. Among these markers, the neutrophil-to-lymphocyte ratio (NLR), lymphocyte-to-monocyte ratio (LMR), and platelet-to-lymphocyte ratio (PLR) have garnered particular attention. These ratios are derived from routine complete blood counts (CBCs) and are relatively easy to calculate, making them convenient tools for assessing the inflammatory status of patients.

The NLR is calculated by dividing the number of neutrophils by the number of lymphocytes, and it has been studied extensively as a marker of inflammation and stress response in various conditions, including cardiovascular diseases, cancers, and infections [12,13]. An elevated NLR has been associated with poor outcomes in several clinical scenarios, reflecting a heightened inflammatory response and immune dysregulation. Similarly, the LMR, calculated by dividing the number of lymphocytes by the number of monocytes, has been explored as a prognostic marker in conditions such as malignancies and inflammatory diseases [14,15]. A lower LMR is often indicative of a more pronounced inflammatory state. The PLR, obtained by dividing the number of platelets by the number of lymphocytes, has also been studied as an inflammatory marker, particularly in cardiovascular and oncological settings, with higher PLR values correlating with worse clinical outcomes.

Despite the widespread use of adenoidectomy and tonsillectomy in clinical practice, there is limited research on the impact of these procedures on systemic inflammation as measured by NLR, LMR, and PLR. Understanding how these markers change in response to surgery could provide valuable insights into the inflammatory processes involved and the potential benefits of these procedures beyond the immediate relief of symptoms.

This study aims to evaluate the changes in NLR, LMR, and PLR in a large cohort of patients undergoing adenoidectomy and/or tonsillectomy. By analyzing preoperative and postoperative values of these inflammatory markers, we seek to determine whether these surgical interventions lead to a measurable reduction in systemic inflammation. This could have significant implications for the management and monitoring of patients undergoing these common ENT procedures.

In addition to providing data on the inflammatory response to adenoidectomy and tonsillectomy, this study also aims to contribute to the growing body of literature on the utility of NLR, LMR, and PLR as biomarkers in clinical practice. By establishing a relationship between these markers and the surgical outcomes of adenoidectomy and tonsillectomy, we hope to highlight their potential role in guiding clinical decision-making and monitoring the postoperative recovery process.

Overall, the findings of this study could lead to a better understanding of the inflammatory dynamics associated with adenoidectomy and tonsillectomy, potentially influencing both the preoperative evaluation and postoperative care of patients undergoing these procedures. The identification of significant changes in NLR, LMR, and PLR post-surgery could also underscore the importance of these markers in the broader context of surgical and medical management, offering clinicians additional tools for assessing patient status and prognosis.

## 2. Materials and Methods

### 2.1. Participants and Study Setting

This study was approved by the Inha University Hospital Ethics Committee and included 980 patients (632 males [64.5%] and 348 females [35.5%]) who visited the ENT outpatient clinic at the Department of Otorhinolaryngology, Inha University Hospital, between August 2000 and August 2024. The preoperative and postoperative test results of patients who underwent surgery were retrospectively analyzed through a review of medical records. Patients were divided into three groups based on their surgical procedure: the adenoidectomy group (33 patients, median age [IQR]: 5.0 [2.0]), the tonsillectomy group (685 patients, median age [IQR]: 5.0 [2.0]), and the adenoidectomy and tonsillectomy group (262 patients, median age [IQR]: 5.0 [2.0]).

### 2.2. Variables and Assessment Measurement

For all patients, changes in white blood cell (WBC) counts, as well as neutrophil, monocyte, lymphocyte, and platelet counts, were obtained from their preoperative and postoperative complete blood cell differentials. The neutrophil-to-lymphocyte ratio (NLR), lymphocyte-to-monocyte ratio (LMR), and platelet-to-lymphocyte ratio (PLR) were calculated and compared within and between the groups. Patients who were lost to follow-up or lacked postoperative complete blood cell counts were excluded from the study.

### 2.3. Statistics

Descriptive statistics (median [IQR, interquartile range]) were used to evaluate the data. After confirming normality with the Shapiro–Wilk test, the Wilcoxon test, the chi-square test, and the Kruskal–Wallis test were used to compare quantitative data and non-normally distributed parameters between groups, with Dunn’s test employed for post hoc analysis. All analyses were performed using GraphPad Prism software V8, and statistical significance was set at *p* < 0.05.

## 3. Results

### 3.1. Participant Characteristics

This study involved 980 patients treated in our clinic, divided into three groups: the adenoidectomy group, the tonsillectomy group, and the combined adenoidectomy and tonsillectomy group. The adenoidectomy group included 33 patients with a median age of 5.0 [2.0] years (range 2–7 years). The tonsillectomy group included 685 patients with a median age of 5.0 [2.0] years (range 1–7 years). The combined adenoidectomy and tonsillectomy group included 262 patients with a median age of 5.0 [2.0] years (range 2–7 years). There was no significant difference in age and sex among the groups (Table 1).

### 3.2. Changes in Hematological Parameters Following Adenoidectomy and Tonsillectomy

**Figure 1:**
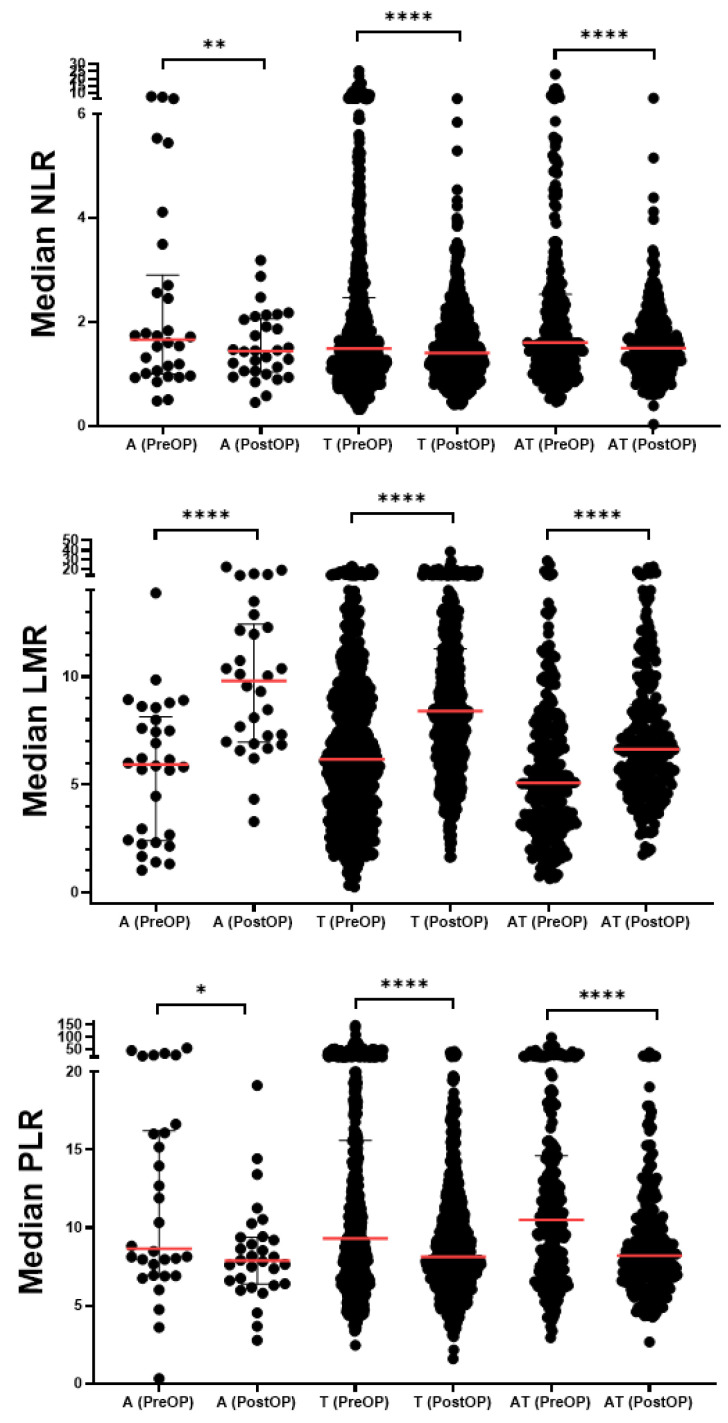
NLR, LMR, and PLR values in the pre-and postoperative groups. The preoperative (Pre-OP) and postoperative (Post-OP) values of neutrophil-to-lymphocyte ratio (NLR), lymphocyte-to-monocyte ratio (LMR), and platelet-to-lymphocyte ratio (PLR) across three surgical groups—adenoidectomy, tonsillectomy, and adenoidectomy + tonsillectomy—are presented. Values are shown as median [IQR] and the median is indicated by a red line. Statistical significance between pre-OP and post-OP values was assessed using the Wilcoxon matched-pairs signed rank test, * *p* < 0.05, ** *p* < 0.01, **** *p* < 0.0001.

Blood analysis results indicated that postoperative neutrophil, postoperative lymphocyte, and postoperative platelet levels were significantly higher compared to their preoperative levels in all groups (*p* < 0.05). Additionally, in the tonsillectomy and adenoidectomy + tonsillectomy groups, postoperative WBC levels were significantly higher compared to preoperative values (*p* < 0.001), whereas no significant difference was observed in the adenoidectomy group. In the adenoidectomy group, postoperative monocyte levels increased, while in the tonsillectomy group, they decreased. However, in the adenoidectomy + tonsillectomy group, no significant changes in monocyte levels were observed (Table 2). In the adenoidectomy, tonsillectomy, and adenoidectomy + tonsillectomy groups, postoperative NLR and postoperative PLR were significantly higher compared to their preoperative values (*p* < 0.05). Additionally, postoperative LMR was significantly lower in all three groups compared to preoperative values, as shown in Table 2 and Figure 1.

## 4. Discussion

This study aimed to evaluate the changes in the neutrophil-to-lymphocyte ratio (NLR), lymphocyte-to-monocyte ratio (LMR), and platelet-to-lymphocyte ratio (PLR) in patients undergoing adenoidectomy and/or tonsillectomy. The results indicate significant postoperative reductions in NLR and PLR, alongside a significant increase in LMR, when compared to their preoperative values. These findings suggest that adenoidectomy and tonsillectomy are associated with a reduction in systemic inflammation, which could have important clinical implications for the management of patients with conditions necessitating these procedures.

### 4.1. Significance of NLR, LMR, and PLR in Clinical Practice

NLR, LMR, and PLR are increasingly recognized as valuable inflammatory markers in various clinical settings, providing insights into the immune response and the overall inflammatory status of patients [11,12,13,14,15]. An elevated NLR is generally considered a marker of systemic inflammation, reflecting an increase in neutrophil counts (which indicate an acute inflammatory response) relative to lymphocyte counts (which are often reduced in the context of stress or inflammation). Similarly, a low LMR suggests a higher inflammatory burden, as it represents a relative increase in monocytes—a key player in chronic inflammation—compared to lymphocytes. The PLR, on the other hand, combines the pro-inflammatory role of platelets with the immune-regulatory function of lymphocytes, with higher PLR values often correlating with worse outcomes in inflammatory and neoplastic diseases [16,17,18].

The significant changes observed in NLR, LMR, and PLR post-surgery in this study suggest that adenoidectomy and tonsillectomy have a profound impact on the inflammatory milieu. The decrease in NLR and PLR, combined with the increase in LMR, indicates a shift towards a less inflammatory state following surgery. This is particularly relevant in the context of diseases like obstructive sleep apnea, recurrent tonsillitis, and chronic adenoiditis, where chronic inflammation plays a central role in pathogenesis and symptomatology [19,20].

### 4.2. Implications for Postoperative Inflammatory Status

The observed reduction in NLR and PLR after adenoidectomy and/or tonsillectomy suggests that these procedures may help mitigate systemic inflammation. Chronic inflammation has been linked to various complications, including cardiovascular disease, metabolic syndrome, and even malignancies [18,21,22]. Therefore, reducing systemic inflammation through surgical intervention may have broader health benefits beyond the immediate relief of airway obstruction and infection control.

The increase in LMR post-surgery is also noteworthy. Lymphocytes play a crucial role in immune regulation and surveillance, and a higher LMR could reflect a more balanced and controlled immune response postoperatively. This shift might contribute to the overall improvement in patient outcomes, potentially reducing the risk of postoperative complications and promoting better recovery.

Moreover, the findings highlight the potential utility of NLR, LMR, and PLR as markers for monitoring surgical outcomes. Regular assessment of these markers could provide clinicians with valuable information about the patient’s inflammatory status, allowing for more personalized postoperative care. For instance, patients with persistently elevated NLR or PLR after surgery might benefit from closer monitoring or additional interventions to address ongoing inflammation.

### 4.3. Comparison with Previous Studies

While limited research has focused specifically on the impact of adenoidectomy and tonsillectomy on NLR, LMR, and PLR, the results of this study are consistent with broader literature that underscores the inflammatory nature of these conditions and the potential for surgical intervention to reduce systemic inflammation [19,23,24]. Previous studies have demonstrated that elevated NLR is associated with worse outcomes in patients with chronic inflammatory conditions and that interventions that reduce inflammation can lead to improvements in these markers.

The findings of this study align with these observations, suggesting that the reduction in systemic inflammation following adenoidectomy and tonsillectomy could be a key mechanism by which these surgeries confer their clinical benefits. By addressing the underlying inflammatory burden, these procedures may not only relieve symptoms but also improve overall health and reduce the risk of long-term complications associated with chronic inflammation.

### 4.4. Clinical and Research Implications

The significant changes in NLR, LMR, and PLR observed in this study have several important clinical and research implications. First, they suggest that these markers could be used as part of the routine evaluation and monitoring of patients undergoing adenoidectomy and tonsillectomy. By assessing these ratios pre- and postoperatively, clinicians could gain valuable insights into the patient’s inflammatory status and the effectiveness of the surgical intervention.

Furthermore, the results of this study underscore the importance of considering the systemic effects of surgical interventions. While adenoidectomy and tonsillectomy are primarily performed to address localized symptoms, the impact of these procedures on systemic inflammation should not be overlooked. This could have implications for patient selection, surgical planning, and postoperative care, particularly in patients with comorbidities that could be exacerbated by systemic inflammation.

From a research perspective, these findings highlight the need for further studies to explore the mechanisms underlying the changes in NLR, LMR, and PLR following adenoidectomy and tonsillectomy. Understanding the pathways through which these surgeries reduce systemic inflammation could lead to new therapeutic strategies aimed at enhancing patient outcomes. Additionally, future research could investigate the long-term effects of these surgeries on systemic inflammation and whether the observed changes in inflammatory markers translate into reduced risks of long-term complications.

### 4.5. Limitations and Future Directions

Despite the strengths of this study, including its large sample size and comprehensive analysis of inflammatory markers, there are several limitations that should be acknowledged. First, the retrospective design of the study may introduce bias, as it relies on the accuracy and completeness of medical records. Additionally, the study did not account for other factors that could influence inflammatory markers, such as comorbid conditions, medications, or lifestyle factors. This study is retrospective, and postoperative blood values were not consistently measured at the same time points for all patients. We believe that future prospective studies are necessary to address this issue.

Future research should aim to address these limitations by conducting prospective studies with more detailed data collection, including information on potential confounders. Moreover, studies with longer follow-up periods could provide insights into the long-term effects of adenoidectomy and tonsillectomy on systemic inflammation and patient outcomes.

## 5. Conclusions

In conclusion, this study demonstrates that adenoidectomy and tonsillectomy are associated with significant reductions in systemic inflammation, as evidenced by changes in NLR, LMR, and PLR. These findings support the use of these markers in monitoring the inflammatory response to surgery and highlight the potential broader health benefits of reducing systemic inflammation through surgical intervention. Further research is needed to fully understand the implications of these changes and to explore the long-term impact of adenoidectomy and tonsillectomy on patient health.

## Figures and Tables

**Table 1:** Demographic characteristics of patients in different surgical groups.

Variable		Adenoidectomy	Tonsillectomy	Adenoidectomy + Tonsillectomy
Sex	Male	22	436	174
	Female	11	249	88
	Total	33	685	262
Age		5.0 [2.0]	5.0 [2.0]	5.0 [2.0]

The demographic characteristics of patients in the adenoidectomy, tonsillectomy, and adenoidectomy + tonsillectomy groups, including sex distribution and median age with IQR, are presented. Statistical significance was assessed using the chi-square test and Kruskal–Wallis test, followed by Dunn’s test for post hoc analysis.

**Table 2:** Hematological parameters and NLR, LMR, and PLR values of the pre- and postoperative patients.

Variable	Group								
	Adenoidectomy			Tonsillectomy			Adenoidectomy + Tonsillectomy		
	Pre-OP	Post-OP	*p*	Pre-OP	Post-OP	*p*	Pre-OP	Post-OP	*p*
WBC (×10^3^/μL)	7.80 [2.86]	6.70 [10.99]	0.3575	8.13 [3.10]	8.36 [4.44]	<0.0001	7.80 [2.86]	8.33 [4.29]	0.0002
Neutrophils (×10^3^/μL)	48.00 [16.55]	59.40 [20.17]	0.0003	48.30 [15.35]	54.70 [25.95]	<0.0001	48.00 [16.55]	59.40 [20.17]	<0.0001
Lymphocytes (×10^3^/μL)	29.95 [19.95]	40.75 [15.10]	<0.0001	34.10 [23.35]	41.00 [15.30]	<0.0001	29.95 [19.95]	40.75 [15.10]	<0.0001
Monocytes (×10^3^/μL)	5.70 [2.90]	6.00 [2.50]	0.0289	4.90 [2.20]	4.60 [1.80]	0.0008	5.70 [2.90]	6.00 [2.50]	0.1300
Platelets (×10^3^/μL)	296.5 [106.5]	332.0 [91.3]	0.0115	305.0 [107.0]	331.0 [105.0]	<0.0001	296.5 [106.5]	332.0 [106.5]	<0.0001
NLR	1.60 [1.38]	1.50 [0.81]	0.0070	1.49 [1.43]	1.40 [0.85]	<0.0001	1.60 [1.38]	1.50 [0.81]	<0.0001
LMR	5.08 [4.34]	6.64 [3.65]	<0.0001	6.17 [5.35]	8.41 [5.01]	<0.0001	5.08 [4.34]	6.64 [3.65]	<0.0001
PLR	10.50 [7.30]	8.21 [3.86]	0.0178	9.32 [8.80]	8.14 [4.09]	<0.0001	10.50 [7.30]	8.21 [3.86]	<0.0001

The hematological parameters, including WBC, neutrophils, lymphocytes, monocytes, platelets, and their derived ratios (NLR, LMR, PLR) before (pre-OP) and after (post-OP) surgery for the adenoidectomy, tonsillectomy, and adenoidectomy + tonsillectomy groups are presented. Data are shown as median [IQR]. The *p* values were obtained using the Wilcoxon matched-pairs signed rank test for comparisons between pre-OP and post-OP values.

## Data Availability

The data is available upon request from the corresponding author.
